# Quark-Type Cheese: Effect of Fat Content, Homogenization, and Heat Treatment of Cheese Milk

**DOI:** 10.3390/foods10010184

**Published:** 2021-01-18

**Authors:** Sofia Lepesioti, Evangelia Zoidou, Dionysia Lioliou, Ekaterini Moschopoulou, Golfo Moatsou

**Affiliations:** Laboratory of Dairy Research, Department of Food Science and Human Nutrition, Agricultural University of Athens, Iera Odos 75, 118 55 Athens, Greece; sofialepesioti@gmail.com (S.L.); ezoidou@aua.gr (E.Z.); dionisia.lioliou@gmail.com (D.L.); catmos@aua.gr (E.M.)

**Keywords:** Quark-type cheese, cow cheese milk homogenization, cheese milk heat treatment, reduced-fat cheese, sugars and organic acids, proteolysis indices, texture profile analysis, whey protein denaturation

## Abstract

The effect of homogenization and fat reduction in combination with variable heating conditions of cow milk on the characteristics of Quark-type cheese were investigated. The mean composition of full-fat cheeses was 71.96% moisture, 13.95% fat, and 10.31% protein, and that of its reduced-fat counterparts was 73.08%, 10.39%, and 12.84%, respectively. The increase of heat treatment intensity increased moisture retention and improved the mean cheese protein-to-fat ratio from 0.92 to 1. Homogenization increased the moisture and protein retention in cheese, but the effect was less intense for milk treated at 90 °C for 5 min. The extended denaturation of whey proteins resulted in harder, springier, and less cohesive cheese (*p* < 0.05). Treatment of milk at 90 °C for 5 min resulted in higher residual lactose and citric acid and lower water-soluble nitrogen contents of cheese (*p* < 0.05); the latter was also true for homogenization (*p* < 0.05). Storage did not affect the composition and texture but decreased galactose and increased citric acid and soluble nitrogen fractions (*p* < 0.05). In conclusion, heat treatment conditions of milk that induced a considerable denaturation of β-lactoglobulin and left a considerable amount of native α-lactalbumin was adequate for the manufacture of a “clean-label” Quark-type cheese, whereas homogenization was more effective for full-fat cheese.

## 1. Introduction

Fresh, spreadable cheese varieties with paste-like consistency such as Quark-type and Cream cheese come mainly from combined acid-rennet curds, in which hydrated acid casein gel particles are dispersed in whey [1,2]. According to Codex Alimentarius [3], cheeses of the category “Cream cheese” are for the most part intended for direct consumption, can spread and mix readily with other foods, and contain at least 22% dry matter and 67% moisture on a fat-free basis. A brief literature outline of the effects and mechanisms observed in combined acid–rennet curd/cheese due to interventions on cheese milk such as fat reduction, heating, and homogenization, which are related to present study, is given below.

To counteract the effects of fat reduction on the paracasein matrix and on the subsequent ripening course, modifications are applied in (i) the treatment of reduced-fat cheese milk, i.e., heating conditions, homogenization, fat mimetic,s or replacers, and (ii) the cheesemaking conditions, i.e., starter mixture, curdling, cutting, scalding, in-vat acidification, salting [4,5]. Milk heat treatment can be a means to control the texture and yield of dairy products, e.g., heating at 95 °C for 15 min or at 90 °C for 10 min increases Quark yield, moisture, and firmness [6,7]. Treatment more intense than typical, low pasteurization causes an in situ denaturation of whey proteins that form soluble aggregates and complexes with casein micelles, which in turn reduce syneresis and moisture loss from the curd [8,9,10]. The complexation of casein with whey proteins denatured by the heating of skim milk at 80 °C for 30 min hinders the rennet action on κ-casein and the concomitant aggregation of rennet-altered micelles. Therefore, smaller pores, lower permeability, lower whey separation, less sensitivity to large-scale arrangements, and higher firmness are observed in the resultant combined acid–rennet gels compared to unheated counterparts [11]. Whey proteins denatured by the heating of milk at 82 °C and 90 °C for 5 min participate in the gel formation of Quark. As a result, a regular microstructure with large protein aggregates, branched clusters, and numerous small pores is developed opposite to the larger pores of pasteurized milk at72 °C for 16 s [12]. Similarly, an increase of pasteurization temperature from 65 to 75 °C for 30 min increased substantially the moisture and yield of a spreadable goat milk [13]. However, it has been reported that the increase of milk heating temperature from 80 to 90 °C for 5 min has no significant effect on syneresis and water-holding capacity and decreases the firmness and stickiness of Quark-type gels [14].

The homogenization of cheese milk or cream under specific conditions has been proposed as a means to increase the moisture and improve the texture and heat-induced functionality of reduced- or low-fat cheeses [4,9]. The reduced size of homogenized milk fat globules (MFG) and the casein absorption onto their surface enables them behave as pseudo-protein particles interacting with casein micelles during coagulation opposite to the MFG of the unhomogenized milk, which are embedded into the paracasein matrix [15,16]. Consequently, the fusion of the paracasein micelles and whey removal are not favored, resulting in reduced gel strength and poor syneresis, high moisture, and increased fineness of the homogenized milk curds that can deviate the ripening course and functionality [4,9]. The homogenization of cheese milk is applied for the manufacture of soft, high-fat, acid-rennet curd Quark or Cream cheese. It counteracts creaming during the long curdling period, reduces fat loss in the whey, and increases the effective protein concentration. Moreover, the participation of homogenized MFG in gel formation contributes to the development of a homogeneously soft and smooth texture on subsequent acidification without wheying off or drying [1,4]. In brief, the following mechanisms have been suggested [17,18]. Ηomogenization at 20 MPa, at normal milk pH 6.7, decreases the ζ-potential of new homogenized MFG that becomes similar to that of casein micelles that are absorbed onto their surface. The protonation of phosphate and carboxylic residues due to acidification decreases further the ζ-potential of both new MFG and caseins to −2.3 and −4.3 mV, respectively whereas in unhomogenized milk, it is −6.6 mV. Furthermore, the replacement of stabilizing highly structured glycocalyx of the native milk fat globule membrane (MFGM) by the more flexible casein and the decrease of electrostatic repulsions at pH 4.5 enhance the hydrophobic interactions between new MFG and casein micelles. Since casein micelles are present at both sides of interaction, homogenized MFG become active fillers [18]. Heating corresponding to an 80% denaturation of total whey proteins reduces further the ζ-potential of the casein micelles and homogenized MFG because denatured whey proteins are complexed onto the casein surface or co-absorbed with caseins onto the homogenized MFGM [17]. Moreover, additional strong hydrophobic interactions between denatured whey proteins result in stranded protein structures and in stiffer and more connected acid gel compared to their unheated homogenized counterpart [17].

The effects of milk homogenization or fat reduction on the physical properties of Cream cheese have been reported. The increase of homogenization pressure from 0 to 25 MPa of milk with protein-to-fat (P/F) ratio 0.97 can decrease approximately five times the D_4,3_ and ten times the D_3,2_ descriptors of particles and increase significantly the firmness, spreadability, and viscosity of medium-fat Cream cheese; at 100 MPa, no further significant differentiation is observed [19]. It has been shown that homogenization at 30 MPa destabilizes acidified whey protein water/oil emulsions when there are not enough non-denatured whey proteins to cover the interface [20]. Homogenization at 15 MPa decrease by eight times the D_4,3_ and more than ten times the D_3,2_ of particles in Cream cheese with a P/F ratio of 0.33, resulting in higher consistency or stiffening of the gel network. Interestingly, the protein content of the bulk phase not absorbed onto the homogenized MFG decreased by seven times [21]. The additional whey protein denaturation caused by outlet heating of post-homogenization of an acidified Cream cheese model enhanced protein-mediated interactions between fat globules, resulting in higher firmness and lower spreadability [22]. The higher hardness, elasticity cohesiveness, and spreadability of full-fat compared to low-fat commercial Cream cheeses have been assigned to the high numbers of homogenized MFGs that counteract the higher protein content of the latter [23]. The decrease of milk fat results in larger particles in the cheese matrix; i.e., the mean particle size in Cream cheese with low-0.5%, medium-5.5%, and high-11.6% fat content has been found at 18.2, 11.3, and 8.1 μm, respectively [24]. The physical properties of commercial Cream cheeses with different fat contents are largely shaped by the effect of stabilizers on their microstructure; however, low fat content and high moisture are consistent with less structured and more spreadable products [25].

Soft, acid cheeses with paste-like consistency are manufactured in various regions of Greece from cow, ewe, goat milk, or their mixtures. They result from the rennet and/or acid coagulation of rather high heat-treated, full-fat, unhomogenized milk. The next processing steps are acidification/curdling, draining by means of cloth filter bags, gentle homogenization by hand, and often salting of the drained curd and a very short or no ripening period; no stabilizers or other additives are used. Differences in the cheesemaking protocols shape a variety of spreadable cheeses, with high moisture (60–75%) and fat on a dry basis (40–55%), rather low salt (1–1.8%) and pH ≅ 4.5; some of them are protected denomination of origin (PDO) cheeses, i.e., Galotyri, Katiki, Piktogalo Chanion, Anevato, Kopanisti, and Xygalo Siteias [26,27,28,29,30].

Considering the consumers’ interest in this cheese type and the current demand for reduced-fat and reduced-energy dairy products, the present study was undertaken to investigate the effect of homogenization and fat reduction in combination with variable heat treatments of cow milk on the characteristics of Quark-type cheese.

## 2. Materials and Methods

### 2.1. Milk Treatments and Analyses

Cheese milk homogenization and continuous heat treatments by means of tubular heat exchanger were carried out in the laboratory heating system HT220 HTST/UHT (OMVE Lab & Pilot Equipment, 3454 MZ De Meern, The Netherlands) equipped with an in-line two-stage homogenizer (Twin Panda, Gea Niro Soavi, Type NS2002H). In one experimental day, raw cow milk from the premises of Agricultural University of Athens was divided in two parts. The first part (milk UH) was heated in the laboratory heating system at 68 °C (1), 73 °C (2), 78 °C (3), 85 °C (4), and 100 °C (5) for 16 s and cooled down at 30 °C. In addition, a portion of milk was heated under batch conditions in an open vat at 90 °C for 5 min (6) and then cooled immediately in an ice bath. The second part was similarly heat treated (conditions 1–6) but after in-line two-stage homogenization at 25 and 5 MPa (milk H). The next day, the same treatments were applied in reduced-fat (RF) milk that was a mixture of raw full-fat (FF) milk and milk skimmed by means of a cream separator. The protein-to-fat (P/F) ratio of RF milk increased to 1.62 from 0.95 of FF, which corresponded to a 38% fat reduction. In each experimental day, 12 milk portions were prepared and along with the two controls (H and UH, not heat-treated) resulted in 14 milk samples. The two-day set of experiments was performed in triplicate.

The gross composition of milk samples was estimated by Milkoscan-FT120 134 (Foss, Hilleroed, Denmark), titratable acidity by means of the Dornic method and pH by means of a pHmeter (WMW Multiline Multi 3420, Fisher Scientific, Loughborough, Leicestershire, UK). Assessment of the heat denaturation of major whey proteins α-lactalbumin (α-la) and β-lactoglobulin (β-lg) was based on their residual content in the pH 4.6 soluble fraction of milk, which was analyzed by the RP-HPLC method, as described by Sakkas et al. [31].

### 2.2. Cheesemakings

Based on the results for whey protein denaturation, four of the above-mentioned heat treatments—that is, 2, 4, 5, and 6—were selected for the treatment of cheesemilk utilized for the manufacture of Quark-type combined curd cheeses. In one experimental day, unhomogenized (UH) and homogenized (H) FF milk portions were heated at 73 °C (2), 85 °C (4), and 100 °C (5) for 16 s and at 90 °C for 5 min and then cooled down to 30 °C as previously described. Ten kg milk from each homogenization–heating combination were collected in a sterilized container, and at 25 °C, 1.8% dry salt was added. Subsequently, inoculation by means of a commercial starter took place (DVS MO-10-Chr. Hansen Holding A/S, Hoershom, 94 Denmark). Then, calf rennet powder with clotting activity 1:100,000 was added at a ratio of 10 mg per L of milk. After gentle stirring, the inoculated milk was left for approximately 18 h at 18–20 °C for acidification to pH 4.6 and curd formation. The curd cut into 2 × 2 × 2 cm cubes was transferred into cloth filter bags for draining at 18–20 °C for approximately 20 h. Then, the fresh cheese was weighed for the estimation of yield, homogenized gently by hand, packed in sterilized containers, and stored at 4 °C. Within the same week, the same experimental cheese makings were carried out, using RF cheese milk.

In each experimental day, 8 cheese makings were carried out. The FF-RF set of experimental cheese makings was performed in triplicate.

### 2.3. Cheese Analyses

Cheeses were analyzed on 5 and 20 day of storage; in total, 96 cheese samples were analysed.

Gross composition of cheeses was estimated by FoodScan-Dairy Near Infrared (NIR) analyzer (Foss, Hilleroed, Denmark). Cheese pH was determined in a dispersion of cheese in water at 1:1 ratio by means of a pH meter. Cheese titratable acidity was estimated in a filtrate of a cheese dispersion; ten g cheese dispersed in 92 mL of distilled water were filtered with coarse filter paper, and the acidity of 15 mL of the filtrate was determined using N/10 NaOH and phenolphalein.

Residual sugars and organic acids, i.e., lactose, glucose, galactose, citric acid, and lactic acid were determined by HPLC by means of the Aminex HPX-87H column (Biorad Inc., Hercules, CA, USA) [32,33]. Twenty g of cheese were diluted to 100 mL with Biggs-Szijarto solution i.e., 25 g zinc acetate dihydrate, 12.5 g phosphotungstic acid monohydrate, and 20 mL glacial acetic acid in 200 mL. After mixing and standing for 10 min, the cheese dilution was filtered with Whatman No. 1 filter paper. One mL of filtrate was mixed with 100 μL perchloric acid (70%) and after an overnight stay at 4 °C, the mixture was centrifuged at 10,000× *g* for 30 min at 4 °C. The supernatant was filtered with 0.45 μm before HPLC analysis; the quantification was based on the respective standard curves.

Texture profile analysis (TPA) was carried out in duplicate at 15 °C using a 5 kg load cell and a 35 mm plunger by means of the double bite test [34,35] using a Shimadzu Testing Instrument AGS-500 NG (Shimadzu Corporation, Kyoto, Japan).

The nitrogen (N) content of cheese (total N, TN), the water-soluble nitrogen (WSN), and the nitrogen soluble in 12% trichloroacetic acid (TCAN) were determined by the Kjeldahl method, as described by Moschopoulou et al. [36]. In brief, 30 g of cheese were homogenized with 90 g of distilled water using Ultra Turrax (IKA Works, Inc., Wilmington, NC, USA) at 9500 rpm for 2 min. After 60 min at 40 °C, the cheese dispersion was re-homogenized under the same conditions followed by centrifugation at 3000× *g*, for 30 min, at 6 °C. The supernatant filtered through Whatman No. 1 filter paper was the WSN fraction. For the preparation of TCAN fraction, 75 mL of WSN were mixed with an equal volume of 24% (*w*/*v*) trichloroacetic acid (TCA), held overnight at 4 °C, and then filtered through Whatman No. 1 filter paper.

Organoleptic evaluation of cheeses at 20 day of storage was performed by a panel of four experienced laboratory staff members. Cheeses coded by three-digit code were randomly presented in the panel for the evaluation of appearance, texture, and flavor using a scale from 0 to 10 points. The total organoleptic score—expressed as percentage—was the sum of the appearance, texture, and flavor scores multiplied by 1, 4, and 5, respectively. Moreover, the panel members noted the existence of specific defects, such as non-homogenous color, spoiled surface, dryness, stickiness, lack of spreadability, excessive acidity and saltiness, bitterness, metallic taste, or yeast flavor.

### 2.4. Statistical Analysis

The effects of homogenization, fat reduction, heat treatments, and their interactions on milk and cheese characteristics were assessed by multifactor ANOVA, whereas the least significance (LSD) method (*p* < 0.05) was applied to test the significant differences. Analysis was performed by means of Statgraphics Centurion XVI (Manugistics, Inc., Rockville, MA 20852, USA).

## 3. Results and Discussion

### 3.1. Characteristics of Cheese Milk

The results of milk analyses are presented in Table 1, which are grouped by the three experimental factors. No significant effects of the interactions were observed, with the exception of residual α-lactalbumin (α-la), which was affected by the interaction of fat content and heat treatment. According to multifactor ANOVA, the effect of homogenization on the compositional parameters and residual whey proteins of cheese milk was limited to acidity, which can be assigned to the required additional time and treatments. As expected, the fat reduction statistically significantly (*p* < 0.05) affected the compositional parameters of cheese milk. In fact, the protein-to-fat ratio increased from 0.95 in full-fat (FF) to 1.62 in reduced-fat (RF) milk. It is evident (Table 1) that the significant effect (*p* < 0.05) of the heat treatment on the compositional parameters of milk was due to the significantly different (*p* < 0.05) milk 6, which was heated under batch conditions in a more or less open container at 90 °C for the rather long period of 5 min; under such conditions, a limited evaporation can occur. The increase of heat load significantly (*p* < 0.05) reduced the native major whey protein content of milk. It is well established that α-la is less heat-sensitive than β-lg; for example, native β-lg was not found at temperatures >100 °C, whereas 43 mg/L residual α-la was estimated at 130 °C [31]. The heating conditions of milk 6 denatured fully β-lg and 75% of α-la. Moreover, the pH of milk 6 was significantly lower (*p* < 0.05), which was apparently due to the equilibria change of calcium phosphate between casein micelles and milk serum [37].

### 3.2. Compositional Characteristics of Cheese

Selected heat treatments 2, 4, 5, and 6 with variable denaturation level of whey proteins were applied in homogenized and unhomogenized full- and reduced-fat cheese milk with the aim of assessing their impact on physicochemical characteristics of the resultant cheeses. Batch heating at 90 °C for 5 min, corresponding to cheese 6 in the present experiments, is close to the traditional practice and is a typical treatment for the manufacture of this cheese type or yoghurt. The mean composition of the experimental cheeses, grouped according to the experimental factors, is shown in Table 2. All the experimental factors affected the composition of the experimental cheeses, except for storage. In fact, storage caused an increase (*p* < 0.05) of acidity expressed as percentage of lactic acid that can be attributed to the catabolism of residual lactose by the starters.

Apparently, the fat reduction of cheese milk significantly (*p* < 0.05) affected *p*< the parameters of Table 2. On average, full-fat (FF) cheeses contained 71.96% moisture, 13.95% fat, 10.31% protein, and 1.26% salt. The respective contents for the reduced-fat (RF) counterparts were 73.08%, 12.84%, 1.45%, and 10.39%. The protein-to-fat ratios were lower than those of the respective cheese milks, i.e., 0.74 and 1.24 for FF and RF cheeses indicating higher protein loss than fat in the whey, during draining.

Homogenization significantly (*p* < 0.05) improved the moisture retention in cheese curd, and consequently, fat and protein contents were reduced. On average, homogenization increased the retention of protein in cheese; the P/F ratio was 0.96, which was higher than the 0.94 of its unhomogenized counterpart. Indeed, the mean protein on dry matter of H cheeses was higher, i.e., 42.42% compared to 41.86% of UH cheese. The mechanisms related to the effect of homogenization with emphasis on acid gels have been concisely presented in the interaction. Opposite to the native, the homogenized MFGs participate in the casein matrix with the interactions being stronger at acidic pH [15,16,18] such as the pH of the cheeses of the present study. These interactions do not favor the fusion of casein micelles; consequently, syneresis is impaired, and cheese moisture increases. The effect of homogenized MFGs on the paracasein matrix has been used as a means to increase the moisture and functionality of rennet-clotted semihard reduced and low-fat cheeses [4]. As mentioned in the Introduction, the homogenization of Cream cheese milk induces an intense decrease of the size of particles within the cheese matrix and reduces the protein amount that is not absorbed onto the homogenized MFGs [19,21].

The importance of milk heat treatment for this type of cheese from combined acid curds formed by the simultaneous acidity development and rennet action on casein has been also highlighted in the Introduction. The participation of denatured whey proteins in the gel and the hindering of rennet action on the casein micelle–whey protein complexes result in curds with smaller pores and lower whey separation [11,12] and finally in spreadable cheese with higher moisture e.g., [13]. A statistically significant increase of cheese moisture and yield caused by a high heat treatment of Quark cheese milk at 90 °C for 10 min compared to typical pasteurization has been reported by Kelly and O’Donelly [7]. They suggested that the level of denaturation of α-la is associated to rheology and texture, while the level of denaturation of β-lg affects moisture and yield. Miloradovic et al. [14] found that the composition of Quark-type cheeses manufactured from cow or goat milk were not affected by the increase of heat treatment of milk from 80 to 90 °C for 5 min, but they report an average increase of yield from 27.4 to 31.3%, respectively, for cow milk cheese. The increase of the intensity of heat treatment (Table 2) improved the P/F ratio apparently due to the inclusion of heat-denatured whey proteins into the curd. The P/F ratio of cheese from the most severely heated cheese 6 was equal to 1, while in cheese 2 from pasteurized milk, it was 0.92. Interestingly, the experimental cheeses made from high heat-treated milk 5 and 6 did not differ in respect to moisture, fat, and protein content, despite the significant differences (*p* < 0.05) in the denaturation level of whey proteins (Table 2).

**Figure 1:**
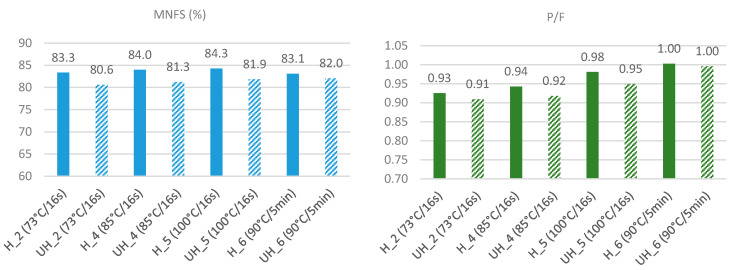
Moisture on non-fat substances (MNFS, %) and protein-to-fat ratio (P/F) of Quark-type cheeses made from homogenized (H) and unhomogenized (UH) milk heat-treated under different conditions.

Significant effects (*p* < 0.05) were observed for the interactions of homogenization–heating on moisture, fat, and protein. The moisture on non-fat substances (MNFS) of cheeses made from homogenized milk (H) was higher than those made from unhomogenized milk (UH). In particular, it was >2.4% higher for the three continuous-type heat treatments 2, 4, and 5 (Figure 1). However, the MNFS difference between H and UH cheese was much lower (1.1%) when the most intense batch treatment at 90 °C for 5 min was applied, indicating that the effect of homogenization is less pronounced when severe denaturation of whey protein takes place. Similarly, the P/F ratio was higher in H cheeses than in UH when they were treated by the continuous heating method, but no difference was observed for the more severe treatment 6 (Figure 1).

Significant (*p* < 0.05) effects of the interaction of homogenization–fat content on cheese moisture and fat were observed. The effect of homogenization on moisture retention was more pronounced in full-fat cheeses. The mean moisture contents of H and UH FF cheeses were 74.08% and 69.84%, and the respective contents for RF cheeses were 74.21% and 71.95%. In particular, homogenization increased the MNFS of FF cheese by 2.7% and that of RF cheese by 1.8%.

### 3.3. Residual Sugars and Organic Acids of Cheese

The concentrations of lactose, monosaccharides, and organic acids were not affected significantly by milk homogenization (Table 3). They were affected by the fat content being significantly higher (*p* < 0.05) in RF cheeses due to the reduced participation of fat into total solids (Table 1). In addition, lactic acid concentration of RF cheese coincided with their significantly (*p* < 0.05) lower pH and higher titratable acidity (Table 1). Heat treatment 6 at 90 °C for 5 min did not affect the monosaccharides and lactic acid but resulted in higher (*p* < 0.05) residual lactose and citric acid contents. The latter could imply a retardation of starter activity, which is not desirable in cheesemaking. During storage, there was a statistically significant (*p* < 0.05) reduction of galactose indicating the slow catabolism from cheese microflora opposite to glucose. The significant (*p* < 0.05) increase of citric acid during storage can be assigned to changes of the paracasein matrix, i.e., proteolysis in Section 3.5—that under the low pH of cheese results in the solubilization of citrate associated with colloidal calcium phosphate [37].

The lactose content of cheese depends for the most part on the cheesemaking conditions that determine its residual amount in the curd. Then, the fermentation of residual lactose and of its subunits glucose and galactose takes place by starters in the first hours or days after manufacture. Moreover, citrate fermentation by particular lactoccoci strains can occur in particular cheese varieties. Pathways of lactose, lactic acid, and citrate catabolism in cheese are presented in detail by McSweeney et al. [38]. The cheesemaking conditions of the present study did not favor the removal of a large quantity of whey, and therefore, a high residual lactose content remained in the cheese mass. Moreover, only a part of it can be catabolized by the starters due to the inhibitory effect of the produced lactic acid on their activity. On average, FF cheeses contained 3.86 ± 0.346 % lactose and 0.720 ± 0.117% lactic acid, whereas the respective contents of RF counterparts were 4.02 ± 0.274 and 1.035 ± 0.310%, which are similar with those reported for this category of cheese. The sum of lactose and lactic acid of skim milk Quark with <1.8% fat and single Cream cheese with 19.5% fat is 3–4% and 3.5%, respectively [1]. Lower contents, i.e., 2.27% lactose and 0.33% lactic acid, reported for Cream cheese with 33.6% fat, can be due to the marked participation of fat in the total solids [1]. A typical gross composition presented by Farkye [2] indicates that low-fat Quark with 82% moisture and pH 4.4–4.6 contains 3–4% lactose, while its full-fat counterpart with 76% moisture and pH 4.5–4.6 contains 2.5–3.5% lactose. A high variability in the lactose content of various artisanal fresh acid-coagulated cheeses with moisture content 44.3–51.3% has been pointed out by Zeppa and Rolle [39]. The lactose content ranged from 2.4 to 22.8 mg per g cheese, the lactic acid ranged from 6 to 27.85 mg/g, glucose was absent, galactose ranged from 0.006 to 4.07 mg/g, and citric acid was from 0.37 to 0.88 mg/g. Papadakis and Polychroniadou [40] reported 14–18 mg lactic acid and 0.12–0.88 mg citric acid per g feta cheese; the respective amounts per g sheep milk yoghurt were 13–16 mg and 1.4–2.3 mg.

### 3.4. Texture Profile Analysis of Cheese

The mean parameters and statistical analysis of texture profile analysis (TPA) of Quark-type experimental cheeses are presented in Table 4 grouped by the experimental factors. Storage exhibited no significant effect (*p* > 0.05) on the texture of cheeses, while the other factors significantly affected (*p* < 0.05) most of these parameters. Moreover, a significant (*p* < 0.05) effect of the interaction homogenization–heating on the hardness was observed.

Gunasekaran and Ak [35] described the TPA parameters as follows. Hardness is the force necessary to attain given information. Adhesiveness is the work necessary to overcome the attractive forces between the surface of the food and surface of other materials with which the food comes into contact. Springiness is the distance recovered by the sample during the time between end of the first bite and start of the second bite. Cohesiveness is the strength of the internal bonds making up the body of the product. Gumminess is the energy needed to disintegrate a semisolid food until it is ready for swallowing. Chewiness is the energy needed to chew a solid food until it is ready for swallowing.

The discussion of our TPA findings in relation to published studies for other paste-like spreadable cheeses is not always adequate, because they are mostly Cream cheese varieties with a rather low protein-to-fat ratio. Quark-type cheeses made from alternative raw materials of dairy origin such as buttermilk [41,42] are not included in the discussion due to the particularities of raw materials that can differentiate the formation and properties of curd.

According to Table 4, the fat reduction resulted in cheeses (RF) with significantly (*p* < 0.05) higher hardness, gumminess, and chewiness and significantly lower adhesiveness and cohesiveness compared to the full-fat counterpart. The effects of fat reduction on the texture profile of rennet-curd cheeses have been reviewed [5,35]. In brief, low-fat rennet–curd cheeses have a more close structure that increases hardness, dryness, graininess, and springiness and decreases adhesiveness and cohesiveness. Moisture increase has the opposite effect on hardness and springiness. However, cheeses of the present experiment resulted from combined curds formed at low pH. Differences in the mechanisms that take place during the formation of cheese curd could affect some textural properties in a different manner, e.g., fat reduction by 10–30% in model cheese analogues based on acid–casein coagulation increased significantly the hardness and adhesiveness [43]. Nguyen et al. [24] report an increase of hardness and adhesiveness when the fat content of Cream cheese is reduced to reach a P/F ratio 0.97, which is much lower than that of the present RF cheeses (Table 2). As presented in the Introduction, the authors attributed these differences to the estimated increase of larger particles in a medium- and low-fat matrix. In addition, they assigned the observed higher stickiness—which is related to adhesiveness—to the increase of protein content due to fat reduction. Moreover, the comparison of the present findings with studies on commercial cheeses is not adequate due to the use of stabilizers in the latter. Stabilizers affect along with fat, moisture, and microstructure the physical properties of this type of cheese [25]; for example, Kealy [44] estimated a lower hardness and adhesiveness and higher cohesiveness for commercial Cream cheeses with stabilizers compared to their full-fat counterparts.

The results of Table 4 for the effect of various heat treatments are in accordance to the brief overview in the Introduction. The heating of cheese milk of combined gels at conditions that induce the denaturation of whey proteins and therefore their complexation with casein micelles increases firmness by decreasing the gel pores, impairing whey separation and improving the regularity of the microstructure [11,12]. The increase of firmness of Quark cheese by heat treatments higher than pasteurization has been reported [6,7]. On the other hand, the lower firmness of Quark-type cheese manufactured from milk batch-heated at 90 °C compared to 80 °C for its 5 min counterpart has been reported [14]; apparently, the batch heating conditions should be taken into consideration for this effect.

The optimum heat treatment for combined curds induces a whey protein denaturation level of 75–80% [1]. Therefore, according to the denaturation level shown in Table 1, batch treatment at 90 °C for 5 min is not adequate for this curd type. The extended denaturation of whey proteins observed in cheese milk 6 (Table 1) resulted in the most hard and springy and the least cohesive cheese (*p* < 0.05). On the other hand, cheese from pasteurized milk was the most soft and adhesive and the least springy (*p* < 0.05). Finally, statistically significant differences (*p* < 0.05) between TPA parameters of heat treatments 4 (85 °C/16 s) and 5 (100 °C/16 s) were scarce. Therefore, the texture of cheeses was not significantly differentiated by heat treatment of milk that induced the denaturation of β-lg and α-la by (i) 37% and 15%, respectively and (ii) by 80% and 25%, respectively.

The mechanisms involved in the formation of acid curd from homogenized milk have been described in the first section of the present article; in conclusion, homogenized MFG acts as active fillers in the acidified casein network [16,17]. The considerable decrease of particles in Cream cheese induced by milk homogenization at conditions similar to ours result in higher firmness, consistency, and spreadability [19,21]. Coutouly et al. [22] report that the post-homogenization of an acidified Cream cheese model resulted in more firm and less spreadable cheese. On the other hand, Brighenti et al. [21] observed that the effect of homogenization counteracted the effect of fat reduction/protein increase in Cream cheese, and increased the hardness, elasticity, cohesiveness, and spreadability of its full-fat counterpart. As shown in Table 4, cheeses from homogenized milk were significantly (*p* < 0.05) softer with significantly lower adhesiveness, gumminess, and chewiness than counterparts from unhomogenized milk. Therefore, cheeses H were less sticky and more easily masticated.

**Figure 2:**
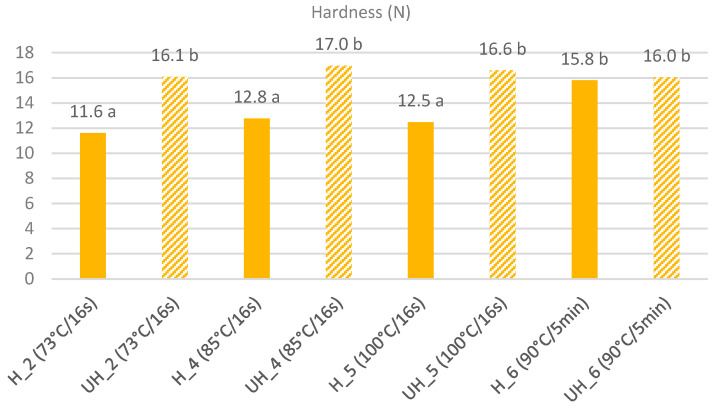
Interaction of homogenization and heating conditions on the hardness of Quark-type cheeses; a,b indicate significant differences (LSD, *p* < 0.05).

Statistical analysis showed that there was a significant (*p* < 0.05) interaction between homogenization and heat treatment, as presented in Figure 2. Similarly, to the above-mentioned weaker effect on the MNFS of cheeses 6, the homogenization of milk did not exhibit any significant effect on cheese hardness when an extended denaturation of whey proteins took place under batch heating at 90 °C for 5 min.

### 3.5. Proteolysis Indices of Cheese

The nitrogen (N) contents of the water-soluble fraction (WSN) and of the fraction soluble in 12% trichloroactic acid (TCAN) of cheeses are presented in Table 5. Storage at low temperature increased significantly (*p* < 0.05) both the WSN and TCAN. On average, WSN expressed as a percentage of total nitrogen (TN) increased from 10.07 ± 1.82 to 11.81 ± 2.35% from 5 to 20 d. Similarly, TCAN/TN increased from 4.49 ± 1.53 to 5.66 ± 1.37%, indicating that secondary proteolysis took place at 4 °C, accumulating small/medium-size peptides and free amino acids [45]. All experimental factors and the homogenization–fat content and homogenization–heating interactions exhibited statistically significant effects (*p* < 0.05) on the proteolysis indices; therefore, they are grouped in Table 5 according to fat content and days of storage.

The increase of the severity of heat treatment significantly (*p* < 0.05) decreased the WSN and WSN/TN ratio in accordance to the level of denaturation of major whey proteins in cheese milk (Table 1). Reduced proteolysis due to the increase of heating of Quark cheese milk has been also reported by Mara and Kelly [46]. Major whey proteins α-la and β-lg are the main WSN constituents of high moisture cheeses. As discussed earlier—in Section 1, Section 3.2 and Section 3.3—heat-denatured whey proteins are complexed with casein micelles and MFGM, thus resulting in reduced nitrogen content of the soluble fraction. Moreover, the significant reduction of WSN observed in both FF and RF cheeses from homogenized milk (H) can be related to complexation with the caseins onto the surface of the homogenized MFGs. Secondary proteolysis in FF and RF cheeses corresponding to the TCAN fraction was not affected in a similar manner by the experimental factors (Table 5). In FF cheeses, the changes of TCAN paralleled those of WSN, decreasing by the increase of heat treatment and by the homogenization of cheese milk. Schematically, secondary proteolysis products included in the TCAN fraction are for the most part attributed to the further degradation of the products of primary proteolysis by the bacterial peptidases. Rennet is expected to be the major factor for primary proteolysis in the cheeses of the present study due to high moisture and low pH [45,46]. However, the low quantity of rennet added in cheese milk should be considered. The access of chymosin to caseins is hindered when they are complexed with denatured whey proteins. The casein matrix of homogenized milks contains also the new MFGs covered by caseins, and consequently, the paracasein matrix includes the homogenized MFGs e.g., [8,9,17]. These effects could be related to the lower proteolytic activity of chymosin post-manufacture, which reduces the primary proteolysis products used as substrates for the secondary changes. However, homogenization significantly (*p* < 0.05) increased the secondary proteolysis of RF cheeses. Since the increase of TCAN is mostly attributed to intracellular bacterial peptidases, a possible effect of RF cheese matrix on the autolysis of the bacterial cells can be taken into account. Similar to our results for RF cheese are the findings of Zamora et al. [47] for a starter-free cheese model with approximately pH 6.7 and moisture 68%, who report that the homogenization of milk at 18–2 MPa decreased WSN/TN but increased free amino groups. The same group [48] studied the effect of homogenization at 18–2 MPa of cheese milk on major proteolytic factors in soft rennet–curd goat milk cheese with 4.6–4.9. They reported that residual chymosin was not affected by homogenization, while the aminopeptidase activity and respective WSN/TN and free amino groups were significantly lower (*p* < 0.05) at the start of ripening.

### 3.6. Organoleptic Evaluation of Cheese

The results of the organoleptic evaluation of cheeses at 20 d of storage are shown in Table 6. The experimental factors and their interactions did not statistically significantly affect the scores with the exception of the significant effect (*p* < 0.05) of fat content on the appearance of cheeses. On storage, no expulsion of whey from cheese mass was observed. Flavor and textural defects were not reported.

The lack of significant differences indicates that the interventions applied in the Quark-type cheese making in the frame of the present study could be acceptable in organoleptic terms despite their above-mentioned profound effects on cheese biochemical and textural characteristics.

## 4. Conclusions

Heat treatments and the homogenization of cheese milk affected significantly (*p* < 0.05) the composition and texture of the experimental Quark-type cheeses. The increase of the intensity of heat treatment increased moisture and protein retention in the curd, and the same was true for homogenization; the effect of the latter was more pronounced in full-fat cheeses. However, the effects were limited when cheese milk was treated under conditions that induced a high level of α-lactalbumin denaturation. Moreover, under these conditions, significantly (*p* < 0.05) higher residual lactose and citric acid contents were observed, implying a retardation of starter activity. Storage at 4 °C did not affect composition and texture but increased proteolysis.

Therefore, heat treatment conditions of cheese milk that induce a considerable denaturation of β-lactoglobulin and leave a considerable amount of native α-lactalbumin were more adequate for the manufacture of this cheese type, whereas homogenization was more effective for full-fat cheeses.

In conclusion, a “clean label”, paste-like, full- or reduced-fat, high-moisture cheese from combined acid–rennet curd can be manufactured by combining physical treatments of cow cheese milk.

## Figures and Tables

**Table 1:** Means of compositional parameters and residual native whey proteins of cheese milk used in the experiments.

Factors	*n*	Fat %	Protein %	Lactose %	TS %	α-la %	β-lg %	pΗ	Acidity %
Homogenization									
H	42	2.65	3.21	4.37	11.34	79.52	63.54	6.76	0.13 ^b^
UH	42	2.67	3.2	4.37	11.34	77.86	62.99	6.74	0.12 ^a^
SE		0.02	0.01	0.01	0.03	0.52	1.18	0.01	0.001
LSD		0.06	0.03	0.03	0.1	1.90	3.37	0.03	0.003
Fat content									
FF	42	3.30 ^b^	3.13 ^a^	4.40 ^b^	11.92 ^b^	77.88	63.62	6.69 ^a^	0.13 ^b^
RF	42	2.03 ^a^	3.28 ^b^	4.34 ^a^	10.76 ^a^	79.49	62.91	6.81 ^b^	0.12 ^a^
SE		0.02	0.01	0.01	0.03	0.52	1.18	0.01	0.001
LSD		0.06	0.03	0.03	0.1	1.89	3.37	0.03	0.003
Heat treatment									
NHT	12	2.61 ^a^	3.20 ^a^	4.33 ^ab^	11.25 ^a^			6.75 ^ab^	0.13
1 (68 °C/16 s)	12	2.60 ^a^	3.14 ^a^	4.29 ^a^	11.13 ^a^	98.60 ^e^	99.52 ^e^	6.77 ^b^	0.12
2 (73 °C/16 s)	12	2.65 ^a^	3.17 ^a^	4.33 ^ab^	11.26 ^a^	95.98 ^e^	99.59 ^e^	6.77 ^b^	0.13
3 (78 °C/16 s)	12	2.65 ^a^	3.18 ^a^	4.34 ^ab^	11.28 ^a^	92.57 ^d^	90.12 ^d^	6.77 ^b^	0.13
4 (85 °C/16 s)	12	2.66 ^a^	3.19 ^a^	4.35 ^b^	11.31 ^a^	84.98 ^c^	67.82 ^c^	6.75 ^ab^	0.12
5 (100 °C/16 s)	12	2.66 ^a^	3.18 ^a^	4.35 ^b^	11.30 ^a^	74.51 ^b^	20.74 ^b^	6.74 ^ab^	0.13
6 (90 °C/5 min)	12	2.80 ^b^	3.36 ^b^	4.59 ^c^	11.84 ^b^	25.49 ^a^	1.82 ^a^	6.70 ^a^	0.13
SE		0.04	0.02	0.02	0.07	0.90	2.01	0.02	0.002
LSD		0.04	0.06	0.06	0.06	3.28	5.84	0.02	0.02

Different letters indicate statistically significant differences (least significant difference (LSD), *p* < 0.05) in the group of means assigned to each experimental factor. SE, standard error; H, homogenized; UH, unhomogenized; FF, full fat; RF, reduced fat; TS, total solids; α-la and β-lg, residual α-lactalbumin and β-lactoglobulin; NHT, not heat-treated.

**Table 2:** Means of compositional parameters of Quark-type cheeses made from unhomogenized and homogenized full- and reduced-fat milk heated under different conditions.

Factors	*n*	Protein %	Moisture %	Salt %	Fat %	pH	Acidity %	Yield %	P/F
Homogenization									
H	48	10.97 ^a^	74.14 ^b^	1.37	11.4 ^a^	4.27	0.58	0.27 ^b^	0.96
UH	48	12.18 ^b^	70.90 ^a^	1.34	12.94 ^b^	4.26	0.59	0.24 ^a^	0.94
SE		0.07	0.12	0.01	0.83	0.009	0.007	0.003	
LSD		0.205	0.363	0.044	0.236	0.027	0.022	0.008	
Fat content									
FF	48	10.31 ^a^	71.96 ^a^	1.26 ^a^	13.95 ^b^	4.28 ^b^	0.56 ^a^	0.28 ^b^	0.74
RF	48	12.84 ^b^	73.08 ^b^	1.45 ^b^	10.39 ^a^	4.24 ^a^	0.61 ^b^	0.24 ^a^	1.24
SE		0.07	0.12	0.01	0.08	0.009	0.007	0.003	
LSD		0.205	0.363	0.044	0.236	0.027	0.022	0.008	
Heat treatment									
2 (73 °C/16 s)	24	11.84 ^b^	71.40 ^a^	1.36	12.92 ^c^	4.26	0.61 ^b^	0.23 ^a^	0.92
4 (85 °C/16 s)	24	11.62 ^ab^	72.29 ^b^	1.35	12.51 ^b^	4.24	0.58 ^ab^	0.25 ^b^	0.93
5 (100 °C/16 s)	24	11.36 ^a^	73.28 ^c^	1.33	11.79 ^a^	4.25	0.58 ^ab^	0.26 ^c^	0.96
6 (90 °C/5 min)	24	11.46 ^a^	73.11 ^c^	1.38	11.47 ^a^	4.28	0.58 ^a^	0.30 ^d^	1.00
SE		0.10	0.18	0.02	0.11	0.01	0.01	0.004	
LSD		0.290		0.063	0.333	0.039	0.031	0.012	
Storage									
5 day	48	11.56	72.51	1.36	12.17	4.26	0.57 ^a^		0.95
20 day	48	11.59	72.53	1.35	12.17	4.26	0.61 ^b^		0.95
SE		0.07	0.12	0.01	0.08	0.009	0.007		
LSD		0.205	0.363	0.044	0.236	0.027	0.022		

Different letters indicate statistically significant differences (LSD, *p* < 0.05) in the group of means assigned to each experimental factor. SE, standard error; H, homogenized; UH, unhomogenized; FF, full fat; RF, reduced fat.

**Table 3:** Means of residual sugars and organic acids (g per 100 g cheese) of Quark-type cheeses made from unhomogenized and homogenized full-fat and reduced-fat milk heated under different conditions.

Factors	Lactose	Glucose	Galactose	Citric Acid	Lactic Acid
Homogenization					
H	3.90	0.177	0.399	0.354	0.833
UH	4.00	0.197	0.493	0.357	0.923
SE	0.043	0.009	0.035	0.086	0.033
LSD	0.119	0.024	0.099	0.024	0.095
Fat content					
FF	3.87 ^a^	0.173 ^a^	0.365 ^a^	0.353	0.720 ^a^
RF	4.03 ^b^	0.200 ^b^	0.527 ^b^	0.358	1.036 ^b^
SE	0.043	0.009	0.035	0.086	0.032
LSD	0.119	0.027	0.099	0.024	0.095
Heat treatment					
2 (73 °C/16 s)	3.82 ^a^	0.191	0.436	0.331 ^a^	0.859
4 (85 °C/16 s)	3.96 ^a^	0.193	0.484	0.361 ^a,b^	0.910
5 (100 °C/16 s)	3.86 ^a^	0.180	0.429	0.360 ^a,b^	0.884
6 (90 °C/5 min)	4.15 b	0.183	0.434	0.369 ^b^	0.859
SE	0.059	0.013	0.490	0.012	0.048
LSD	0.170	0.035	0.140	0.035	0.134
Storage					
5 day	3.91	0.189	0.502 ^b^	0.331 ^a^	0.883
20 day	3.98	0.185	0.389 ^a^	0.379 ^b^	0.872
SE	0.043	0.009	0.035	0.085	0.034
LSD	0.120	0.025	0.099	0.023	0.095

Different letters indicate statistically significant differences (LSD, *p* < 0.05) in the group of means assigned to each experimental factor. SE, standard error; H, homogenized; UH, unhomogenized; FF, full fat; RF, reduced fat.

**Table 4:** Means of the parameters of texture profile analysis of Quark-type cheeses made from unhomogenized and homogenized full- and reduced-fat milk heated under different conditions.

Factors	Hardness	Adhesiveness	Springiness	Cohesiveness	Gumminess	Chewiness
Homogenization						
H	13.16 ^a^	−64.08 ^b^	1.02	0.5	6.52 ^a^	6.70 ^a^
UH	16.41 ^b^	−81.58 ^a^	1.00	0.51	8.34 ^b^	8.42 ^b^
SE	0.34	2.46	0.009	0.007	0.17	0.19
LSD	0.984	6.92	0.026	0.02	0.495	0.536
Fat content						
FF	13.69 ^a^	−68.04 ^b^	1.01	0.51 ^b^	7.01 ^a^	7.11 ^a^
RF	15.88 ^b^	−77.62 ^a^	1.02	0.49 ^a^	7.84 ^b^	8.01 ^b^
SE	0.34	2.46	0.008	0.007	0.17	0.19
LSD	0.984	6.92	0.026	0.02	0.495	0.536
Heat treatment						
2 (73 °C/16 s)	13.84 ^a^	−66.62 ^a^	0.99 ^a^	0.51 ^a,b^	6.95	6.90 ^a^
4 (85 °C/16 s)	14.86 ^a,b^	−78.16 ^b^	1.03 ^b^	0.51 ^a,b^	7.55	7.84 ^b^
5 (100 °C/16 s)	14.53 ^a,b^	−72.39 ^a,b^	1.01 ^a,b^	0.52 ^b^	7.57	7.67 ^b^
6 (90 °C/5 min)	15.91 ^b^	−74.15 ^a,b^	1.02 ^b^	0.48 ^a^	7.64	7.83 ^b^
SE	0.49	3.48	0.01	0.01	0.24	0.26
LSD	1.39	9.79	0.037	0.028	0.7	0.758
Storage						
5 day	14.64	−74.09	1.01	0.52	7.48	7.62
20 day	14.93	−71.06	1.01	0.50	7.37	7.50
SE	0.34	2.46	0.009	0.07	0.17	0.19
LSD	0.984	6.92	0.026	0.02	0.495	0.536

Different letters indicate statistically significant differences (LSD, *p* < 0.05) in the group of means assigned to each experimental factor. SE, standard error; H, homogenized; UH, unhomogenized; FF, full fat; RF, reduced fat.

**Table 5:** Means of proteolysis indices of Quark-type cheeses made from unhomogenized and homogenized full- and reduced-fat milk heated under different conditions.

Index	%WSN		%WSN/TN		%TCAN		%TCAN/WSN	
Days	5	20	5	20	5	20	5	20
	**Full-Fat Cheese (FF)**							
Homogenization								
H	0.166 ^a^	0.194 ^a^	10.29 ^b^	13.11 ^b^	0.061 ^a^	0.086 ^a^	37.52 ^a^	44.04 ^a^
UH	0.180 ^b^	0.208 ^b^	9.79 ^a^	11.54 ^a^	0.086 ^b^	0.103 ^b^	48.05 ^b^	49.61 ^b^
SE	0.0026	0.0045	0.161	0.408	0.0021	0.0025	1.28	1.05
LSD	0.0079	0.0136	0.482	1.22	0.0062	0.0074	3.84	3.15
Heat treatment								
2 (73 °C/16 s)	0.199 ^d^	0.224 ^c^	11.71 ^d^	13.484 ^c^	0.087 ^c^	0.109 ^c^	43.54 ^c^	48.93 ^b^
4 (85 °C/16 s)	0.182 ^c^	0.209 ^b,c^	10.80 ^c^	13.147 ^b,c^	0.085 ^c^	0.105 ^c^	49.59 ^b^	50.76 ^b^
5 (100 °C/16 s)	0.162 ^b^	0.192 ^a,b^	9.33 ^b^	11.671 ^a,b^	0.068 ^b^	0.090 ^b^	42.01 ^b^	46.87 ^b^
6 (90 °C/5 min)	0.148 ^a^	0.180 ^a^	8.32 ^a^	10.997 ^a^	0.054 ^a^	0.074 ^a^	36.01 ^a^	40.73 ^a^
SE	0.0037	0.0064	0.228	0.5781	0.0028	0.0035	1.81	1.48
LSD	0.0112	0.0191	0.683	1.733	0.0090	0.0105	5.42	4.45
	**Reduced-Fat Cheese (RF)**							
Homogenization								
H	0.173 ^a^	0.188 ^a^	9.52 ^a^	9.79 ^a^	0.097 ^b^	0.115 ^b^	53.91 ^b^	64.25 ^b^
UH	0.225 ^b^	0.252 ^b^	10.91 ^b^	12.89 ^b^	0.077 ^a^	0.092 ^a^	36.20 ^a^	38.60 ^a^
SE	0.0090	0.0037	0.423	0.227	0.0055	0.0032	2.18	1.54
LSD	0.0251	0.0110	1.272	0.664	0.0167	0.0098	6.69	4.67
Heat treatment								
2 (73 °C/16 s)	0.219 ^b^	0.253 ^b^	11.21 ^b^	13.04 ^a^	0.095 ^b^	0.106 ^b^	36.96 ^a^	42.03 ^a^
4 (85 °C/16 s)	0.204 ^b^	0.212 ^b^	10.38 ^b^	10.96 ^c^	0.098 ^b^	0.111 ^b^	49.08 ^b,c^	53.54 ^b^
5 (100 °C/16 s)	0.212 ^b^	0.229 ^b^	11.12 ^b^	12.08 ^b^	0.069 ^a^	0.088 ^a^	40.06 ^a,b^	52.25 ^b^
6 (90 °C/5 min)	0.150 ^a^	0.189 ^a^	8.16 ^a^	9.27 ^d^	0.085 ^a,b^	0.108 ^b^	54.11 ^c^	57.88 ^b^
SE	0.0133	0.0052	0.620	0.302	0.0080	0.0043	3.11	2.17
LSD	0.0376	0.0155	1.879	0.966	0.0240	0.0139	9.84	6.61

Different letters indicate statistically significant differences (LSD, *p* < 0.05) in the group of means assigned to each experimental factor. H and UH, homogenized and unhomogenized cheese milk; WSN, water-soluble nitrogen; TCAN, nitrogen soluble in 12% trichloroacetic acid; SE, standard error; LSD, least significance difference.

**Table 6:** Means of organoleptic scores of Quark-type cheeses at 20 d of storage made from unhomogenized and homogenized full- and reduced-fat milk heated under different conditions. Different letters indicate statistically significant differences (LSD, *p* < 0.05) in the group of means assigned to each experimental factor. H and UH, homogenized and unhomogenized cheese milk; FF, full fat; RF, reduced fat; SE, standard error; LSD, least significance difference.

Factors	Appearance 0–10	Texture 0–40	Flavour 0–50	Total Score 0–100
Homogenization				
H	9.04	32.73	39.47	77.94
UH	8.98	33.08	38.96	78.37
SE	0.07	0.50	1.06	2.41
LSD	0.21	1.45	3.02	6.90
Fat-content				
FF	8.84 ^a^	32.78	38.50	79.60
RF	9.18 ^b^	33.04	39.93	76.71
SE	0.07	0.50	1.06	2.41
LSD	0.21	1.45	3.02	6.9
Heat treatment				
2 (73 °C/16 s)	9.00	33.03	37.14	75.23
4 (85 °C/16 s)	8.95	32.56	40.00	79.87
5 (100 °C/5 min)	9.02	33.16	39.07	78.31
6 (90 °C/16 s)	9.06	32.90	40.65	79.22
SE	0.10	0.70	1.46	3.34
LSD	0.29	2.01	4.27	9.50

## Data Availability

The data presented in this study are available on request from the corresponding author.
